# Ideal marker for targeted axillary dissection (IMTAD): a prospective multicentre trial

**DOI:** 10.1186/s12957-023-03147-x

**Published:** 2023-08-19

**Authors:** Jan Žatecký, Oldřich Coufal, Ondřej Zapletal, Otakar Kubala, Markéta Kepičová, Adéla Faridová, Karel Rauš, Jiří Gatěk, Peter Kosáč, Matúš Peteja

**Affiliations:** 1 Department of Surgical Oncology, Masaryk Memorial Cancer Institute, Brno, Czech Republic; 2 Department of Surgery, Silesian Hospital in Opava, Opava, Czech Republic; 3 Faculty of Public Policies, The Institute of Paramedical Health Studies, Silesian University, Opava, Czech Republic; 4 Department of Surgical Oncology, Faculty of Medicine, Masaryk University, Brno, Czech Republic; 5 Department of Surgical Studies, Faculty of Medicine, University of Ostrava, Ostrava, Czech Republic; 6 Department of Surgery, University Hospital Ostrava, Ostrava, Czech Republic; 7 Oncogynecology Centre, The Institute for the Care of Mother and Child, Prague, Czech Republic; 8 Department of Surgery, EUC Clinic Zlín, Zlín, Czech Republic; 9 Tomáš Baťa University in Zlín, Zlín, Czech Republic

**Keywords:** Targeted axillary dissection, Breast cancer, Magnetic seed, Iodine seed, Clip, Carbon suspension

## Abstract

**Background:**

Targeted axillary dissection (TAD) is an established method for axillary staging in patients with breast cancer after neoadjuvant chemotherapy (NAC). TAD consists of sentinel lymph node biopsy and initially pathological lymph node excision, which must be marked by a reliable marker before NAC.

**Methods:**

The IMTAD study is a prospective multicentre trial comparing three localisation markers for lymph node localisation (clip + iodine seed, magnetic seed, carbon suspension) facilitating subsequent surgical excision in the form of TAD. The primary outcome was to prospectively compare the reliability, accuracy, and safety according to complication rate during marker implantation and detection and marker dislodgement.

**Results:**

One hundred eighty-nine patients were included in the study—in 135 patients clip + iodine seed was used, in 30 patients magnetic seed and in 24 patients carbon suspension. The complication rate during the marker implantation and detection were not statistically significant between individual markers (*p* = 0.263; *p* = 0.117). Marker dislodgement was reported in 4 patients with clip + iodine seed localisation (3.0%), dislodgement did not occur in other localisation methods (*p* = 0.999). The false-negativity of sentinel lymph node (SLN) was observed in 8 patients, the false-negativity of targeted lymph nodes (TLN) wasn´t observed at all, the false-negativity rate (FNR) from the subcohort of ypN + patients for SLN is 9.6% and for TLN 0.0%.

**Conclusion:**

The IMTAD study indicated, that clip + iodine seed, magnetic seed and carbon suspension are statistically comparable in terms of complications during marker implantation and detection and marker dislodgement proving their safety, accuracy, and reliability in TAD. The study confirmed, that the FNR of the TLN was lower than the FNR of the SLN proving that the TLN is a better marker for axillary lymph node status after NAC.

**Trial registration:**

NCT04580251. Name of registry: Clinicaltrials.gov. Date of registration: 8.10.2020.

## Highlights

- A prospective multicentre comparative study of three localization markers for pathological lymph node localization.

- First publication comparing iodine seeds, magnetic seeds, and carbon suspension.

- The markers are statistically comparable in complications during marker implantation and detection and marker dislodgement.

- The markers are reliable and safe methods facilitating targeted axillary dissection in breast cancer patients after NAC.

## Introduction

Targeted axillary dissection (TAD) was first described in 2016 by Caudle et al. for improved axillary staging in patients with breast cancer after neoadjuvant chemotherapy (NAC) [1]. TAD consists of sentinel lymph node biopsy and initially pathological lymph node excision. The pathological lymph node must be verified by a core-cut biopsy and marked before the NAC by a reliable marker facilitating subsequent surgical excision due to the possible regression of the lymph node after the NAC.

Several methods for lymph node localization have been described in the literature, including iodine seed localization, magnetic seed localization, and carbon suspension localization. Caudle et al. suggested using a clip followed by a radioactive iodine seed introduction before the surgery [1]. The magnetic seed was recently approved for long-time localisation and the first studies about pathological lymph node localisation have been published confirming magnetic seed as a reliable marker for TAD [2–4]. The next possibility for lymph node marking is an application of carbon suspension also called tattooing [5–7]. The main difference is that carbon suspension is detected visually during the surgery, but iodine seed and magnetic seed are detected by a probe.

Although some authors have published experiences with the above-mentioned markers, a prospective comparative multicentre study is missing. The IMTAD study aimed to compare these markers in terms of reliability, accuracy and safety during implantation, marker detection and marker dislodgement.

## Methods

The IMTAD study was designed as a prospective multicentre trial comparing three localisation methods for pathological lymph node localisation for TAD–iodine seed (Advantage™ I-125, Iso Aid LLC, USA) with clip (HydroMARK Breast Biopsy Site Marker, Mammotome, USA), magnetic seed (Magseed®, Endomagnetics Ltd, UK) and carbon suspension (4% solution of carbo adsorbens in normal saline manufactured in local hospital pharmacy). Five surgical departments from the Czech Republic specializing in breast cancer surgery participated in the study during the period from 1.1.2021 to 1.1.2023–Department of Surgical Oncology, Masaryk Memorial Cancer Institute, Department of Surgery, Silesian Hospital in Opava, Department of Surgery, University Hospital Ostrava, Oncogynecology Centre, The Institute for the Care of Mother and Child and Department of Surgery, EUC Clinic Zlín. The study was approved by local Ethics Committees and by the Ethics Committee of the Faculty of Medicine, University of Ostrava. The study was registered on www.clinicaltrials.gov (NCT04580251).

The inclusion criteria were agreement with participation in the IMTAD study with signed informed consent, breast cancer confirmed by biopsy, the indication of NAC, lymph node metastasis confirmed by core-cut biopsy or visible on ultrasound examination (cN+) and localised by clip + iodine seed, magnetic seed or carbon suspension and surgical treatment after NAC in form of TAD. All patients included in the study were discussed by multidisciplinary teams.

All included departments used one of the above-mentioned methods with previous experiences. In the case of iodine seed usage due to radiation safety policy, the lymph node was first localised by a clip, and then before the surgery, iodine seed was implanted near the clip. After the NAC, multidisciplinary teams again discussed patients with information about re-staging and recommend performing TAD and breast tumour operation (mastectomy, breast-conserving surgery). Before the surgery, the sentinel lymph node (SLN) was traced by ^99m^Tc, patent blue was added on an individual basis. Surgery was performed, and the SLN was detected by gamma probe and visually in case of patent blue usage. The localised pathological lymph node was detected by gamma probe (iodine seed), probe Sentimag (magnetic seed), or visually (carbon suspension). Iodine and magnetic seeds in the specimen were routinely intraoperatively verified by specimen mammography. According to histological examination of the lymph nodes from TAD, axillary dissection of level I and II axillary lymph nodes was indicated or omitted. Patients were discharged from the hospital a few days after surgery, outpatient checks were performed.

Observed patients’ parameters were age, side, duration and type of surgery, type of marker localising lymph node, type of marker localising breast tumour (if needed), tumour type, tumour size, TNM classification, tumour grading, number of sentinel lymph nodes and targeted lymph nodes (TLN), time from localisation of the lymph node to surgery, distance from the marker to skin (measured during implantation by ultrasound), complications during the marker implantation or detection (bleeding, marker implantation out of the lymph node, marker dislodgement, failed detection, difficult searching for a targeted node, clip implanted in different node then a seed in case of clip + iodine seed localisation), marker dislodgement (defined as marker finding during the surgery in a position, which ultrasound before the surgery or during the implantation did not described), final histological findings, complications after surgery observed during hospitalization or check-up (seroma, haematoma, lymphoedema, wound infection, wound necrosis, or dehiscence).

The primary outcome was to prospectively compare the reliability, accuracy and safety according to complication rate during marker implantation and detection and dislodgement of three localisation markers used for pathological lymph node localisation with subsequent surgical therapy-TAD. The reliability was defined as the successful completion of TAD with an assessment of complication rate during detection and marker dislodgment. The accuracy was evaluated according to the complication rate during the marker implantation. Safety was defined as the incidence of postoperative complications. The secondary outcome was to compare operation duration according to the used localisation marker and false negativity rate of SLN and TLN. False negativity of SLN/TLN was defined as the proportion of cases when SLN/TLN is negative, but TLN/SLN or other axillary lymph nodes are positive.

Mean values, percentages, and ranges were calculated. Statistical analysis was performed, and *p*-values were calculated using the Fisher exact test, one-way ANOVA test and Kruskal–Wallis test. The normal distribution of data was tested by the Shapiro–Wilk test. The results were considered statistically significant if *p* < 0.05.

## Results

One hundred eighty-nine patients were included in the study meeting inclusion criteria; in 135 patients clip + iodine seed was used, in 30 patients magnetic seed and in 24 patients carbon suspension.

The mean age of patients was 49.4 years (range 26–80 years). The most common tumour type was carcinoma NST in 169 patients (89.4%). According to molecular classification of breast tumours, the most common was luminal B tumour with 61 patients (32.3%) followed by HER2 + with 59 patients (31.2%), triple-negative with 55 patients (29.1%), and luminal A with 14 patients (7.4%). Further cohort characteristics are listed in Table 1.

**Table 1:** Cohort characteristics

Characteristic	Value	Iodine seed + clip	Magnetic seed	Carbon suspension	Total (%)
T stage	T1	27 (20.0%)	15 (50.0%)	12 (50.0%)	54 (28.6%)
	T2	91 (67.4%)	14 (46.7%)	11 (45.8%)	116 (61.4%)
	T3	14 (10.4%)	1 (3.3%)	1 (4.2%)	16 (8.5%)
	T4	3 (2.2%)	0 (0.0%)	0 (0.0%)	3 (1.6%)
cN stage (core-cut biopsy)	N0	1 (0.7%)	5 (16.7%)	2 (8.3%)	8 (4.2%)
	N1	124 (91.9%)	24 (80.0%)	21 (87.5%)	169 (89.4%)
	N2	2 (1.5%)	1 (3.3%)	0 (0.0%)	3 (1.6%)
	N3	8 (5.9%)	0 (0.0%)	1 (4.2%)	9 (4.8%)
ypN stage (post-surgery)	N0	84 (62.2%)	12 (40.0%)	10 (41.7%)	106 (56.1%)
	N1	46 (34.1%)	14 (46.7%)	13 (54.2%)	73 (38.6%)
	N2	3 (2.2%)	3 (10.0%)	1 (4.2%)	7 (3.7%)
	N3	2 (1.5%)	1 (3.3%)	0 (0.0%)	3 (1.6%)
M stage	M0	133 (98.5%)	30 (100.0%)	24 (100.0%)	187 (98.9%)
	M1	2 (1.5%)	0 (0.0%)	0 (0.0%)	2 (1.1%)
Tumour type	NST	128 (94.8%)	22 (73.3%)	23 (95.8%)	173 (91.5%)
	Lobular others	2 (1.5%)	6 (20.0%)	1 (4.2%)	9 (4.8%)
		5 (3.7%)	2 (6.7%)	0 (0.0%)	7 (3.7%)
Grading	G1	2 (1.5%)	8 (26.7%)	5 (20.8%)	15 (7.9%)
	G2	50 (37.0%)	15 (50.0%)	15 (62.5%)	80 (42.3%)
	G3	83 (61.5%)	7 (23.3%)	4 (16.7%)	94 (49.7%)
Type of breast surgery	MAE	58 (43.0%)	7 (23.3%)	8 (33.3%)	73 (38.6%)
	BCS	77 (57.0%)	23 (76.7%)	16 (66.7%)	116 (61.4%)
		135 (100.0%)	30 (100.0%)	24 (100.0%)	189 (100.0%)

*MAE* Mastectomy, *BCS* Breast-conserving surgery

The complications during the marker implantation occurred once in carbon suspension (4.2%) and once in clip + iodine seed (0.7%), in magnetic seed, any complication was not reported (*p* = 0.263). Marker dislodgement was reported in 4 patients with clip + iodine seed localisation (3.0%), dislodgement did not occur in other localisation methods (*p* = 0.999). Complications during peroperative marker detection occurred in 16 patients with clip + iodine seed localisation (11.9%) and in 2 patients with carbon suspension (8.3%), any complication was reported in magnetic seed localisation (*p* = 0.117). The identification rate of the TLN was 96.3% for clip + iodine seed and 100% for magnetic seed and carbon suspension (*p* = 0.792). Further information is listed in Table 2.

**Table 2:** Complications during the marker implantation and detection, marker dislodgement, identification rate

	**Complications during implantation**	Marker** dislodgement**	**Complications during detection**	**IR**
Clip + iodine seed	1 (0.7%)	4 (3.0%)	16 (11.9%)	96.3%
	bleeding with the application of local haemostatics (1; 0.7%)	seed found dislocated in an adipose tissue (4; 3.0%)	clip not found peroperatively (10; 7,4%), seed migration (4; 3.0%), seed implanted in different lymph node then clip (2; 1.5%)	
Magseed	0	0	0	100%
Carbon suspension	1 (4.2%)	0	2 (8.3%)	100%
	application outside the lymph node (1; 4.2%)		difficult searching for a marked node (2; 8.3%)	
*p value*	*0.263*	*0.999*	*0.117*	*0.792*

The information about the depth of marker placement was present in 167 patients. The mean depth was 29.3 mm in clip + iodine seeds (minimum of 9 mm, maximum of 80 mm), 25.4 mm in carbon suspension (18–33 mm) and 13.8 mm in magnetic seed (7–25 mm). The mean time of marker deposition in pathological lymph node was 148.3 days in carbon suspension and 138.5 days in magnetic seed. Iodine seed was inserted the same day as surgery (mean time 0.0 days), and the pathological lymph node was localised before NAC by a clip, so the mean time of clip deposition was 186.7 days.

The operation time was evaluated according to the type of marker used. Patients with bilateral surgery, with breast reconstruction subsequent to the cancer operation and with axillary dissection of level I and II axillary lymph nodes during the same surgery were excluded from the analysis (*n* = 62), so the subcohort consists of 127 patients. The shortest mean operation times were in clip + iodine seeds (49.7 min for BCS; 76.8 min for MAE). The magnetic seed operation times were 57.1 min. for BCS and 74.5 min. for MAE and with carbon suspension the operation times were 69.2 min. for BCS and 80.0 min for MAE. The statistical evaluation did not reveal statistically significant difference between MAE groups (*p* = 0.895), but revealed significant difference between BCS groups (*p* = 0.006).

The mean number of harvested sentinel lymph nodes was 2.8, the median was 2 sentinel lymph nodes with a minimum of 1 and a maximum of 10 SLN. The mean number of SLN was 2.0 in carbon suspension, 2.8 in clip + iodine seed, and 3.1 in magnetic seed. The mean number of all lymph nodes harvested during TAD (targeted and sentinel lymph nodes) was 2.3 in carbon suspension, 3.2 in clip + iodine seed, and 3.5 in the magnetic seed. TLN was different from a sentinel lymph node in 45 (23.8%) patients, 5 patients with magnetic seed (16.7%), 5 patients with carbon suspension (20.8%), and 35 patients with clip + iodine seed localisation (25.9%). The false-negativity of SLN was observed in 8 patients, the false-negativity of TLN was not observed at all, and true positivity of SLN and TLN was observed in 13 and 21 cases, respectively. The false-negativity rate from the subcohort of ypN + patients for SLN is 9.6% and for TLN 0.0%. Further information is listed in Table 3.

**Table 3:** The histological findings in a subcohort of patients with a sentinel lymph node different from a marked lymph node with false-negativity rates

	**SLN**	**TLN**
Negative	31 (68.9%)	23 (51.1%)
ITC	1 (2.2%)	1 (2.2%)
Micrometastasis	2 (4.4%)	6 (13.3%)
Macrometastasis	11 (24.4%)	15 (33.3%)
False-negative	8 (4 micrometastasis, 4 macrometastasis)	0
True-positive	13	21
False-negativity rate for micrometastasis and macrometastasis (from the ypN + subcohort, *n* = 83)	9.6%	0.0%
False-negativity rate for macrometastasis only (from the ypN + subcohort, *n* = 83)	4.8%	0.0%
**Total**	45 (100%)	45 (100%)

*SLN* Sentinel lymph node, *TLN* Targeted lymph node, *ITC* Isolated tumour cells

TAD was not possible to perform in 7 patients with clip + iodine seed localisation (5.2%). The reasons were dislocation of a clip with the inability to find it in 2 patients (1.5%), non-detectable SLN in 2 patients (1.5%) and localisation of lipomatous axillary tissue instead of the lymph node in 3 patients (2.2%). Axillary dissection of level I and II (ALND) was performed in all patients from this subgroup. In 3 patients (2.2%), the iodine seed was placed in another lymph node than clip, but both lymph nodes were excised, so TAD was finished without ALND. In magnetic seed and carbon suspension localisation TAD was performed in all cases (*p* = 0.472).

Axillary dissection of level I and II was performed in 74 patients (39.2%) during the same operation or as a particular surgery. The mean number of harvested axillary lymph nodes during ALND was 10.0 with a minimum of 2 and a maximum of 23 lymph nodes. The most common indication for ALND was the presence of macrometastasis in the sentinel lymph node or TLN (58 out of 74 patients; 78.4%).

The incidence of postoperative complications in the cohort was 14.8%. The most common complications were seroma formation (5.8%) and haematoma (3.7%) in the wound. The comparison between the three studied localisation methods didn´t prove statistical significance in seroma formation (*p* = 0.074) or haematoma incidence (*p* = 0.621). Other complications such as wound infection, necrosis, or dehiscence were present only in one or two patients. The incidence of postoperative complications in the subgroup with TAD only (without ALND) was 7.8% with mostly seroma and haematoma in the wound, but there was also one patient with lymphoedema after TAD (0.9%).

## Discussion

To our knowledge, this is the first published prospective multicentre study comparing three localisation markers for TAD worldwide. According to the literature, axillary staging with TAD could spare up to 41% of patients an axillary dissection of level I and II with the benefit of lower morbidity after surgery [8]. Apart from TAD, some authors also published studies about axillary staging after NAC in the form of TLNB (targeted lymph node biopsy = MLNB = marked lymph node biopsy) without sentinel lymph node biopsy [9, 10]. Swarnkar et al. performed a systematic review of publications about TAD and TLNB and concluded, that both methods are feasible with an acceptably low false-negativity rate–5.18% for TAD and 6.28% for TLNB [11]. Song et al. performed a systematic review and meta-analysis with a similar conclusion–TLNB has a FNR of 5.5% [12]. In most patients the targeted lymph node is also the SLN, but according to the analysis by Coufal et al., 41% of patients with TAD have the SLN different to the targeted lymph node [13]. Our analysis confirmed, that the FNR of the TLN was lower than the SLN (0.0% and 9.6%) proving that the targeted lymph node is a more accurate marker of axillary lymph node status after NAC than the SLN. The common condition for successful TAD or TLNB is a reliable marker for pathological lymph node localisation. Unfortunately, most markers used for lymph node localisation are primarily produced for breast lession localisation; therefore, we suggest that manufacturers should produce lymph node-specific markers.

**Fig. 1:**
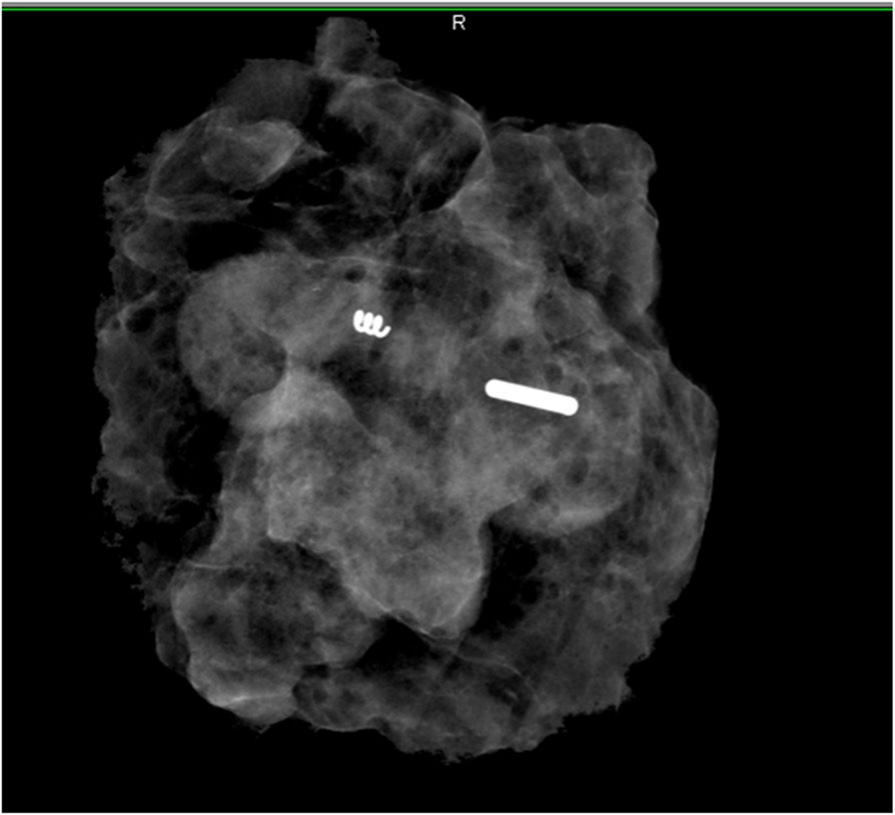
Specimen X-ray of the iodine seed and clip introduced in the lymph node (Masaryk Memorial Cancer Institute)

Each localisation method studied in our trial has its pros and cons. The main advantage of iodine and magnetic seed is a precise localisation by a specific probe, but the common disadvantage is a higher price of markers. The iodine seed detection could be more intuitive for surgeons experienced in sentinel lymph node biopsy using ^99m^Tc [11]. The drawbacks of iodine seed are mainly radioactivity and the need for another marker usage due to the long time from localisation of pathological lymph node to excision, so the patients underwent two localisations instead of one. The iodine seed is approved for implantation for 30 days only according to a manufacturer, but long-term implantation was also studied [9]. Donker et al. presented a study with iodine seed placed in a lymphatic node for a median of 17 weeks with a range of 9–31 weeks with all seeds detected by a probe and surgically excised [9]. In the Czech Republic is the maximum time from iodine seed insertion to the surgery 30 days due to radiation safety policy, so we used a clip in our study for long-term lymph node marking and then the iodine seed was implanted before the surgery (Fig. 1). The same protocol was used by Caudle et al. in the first study about TAD [1].

**Fig. 2:**
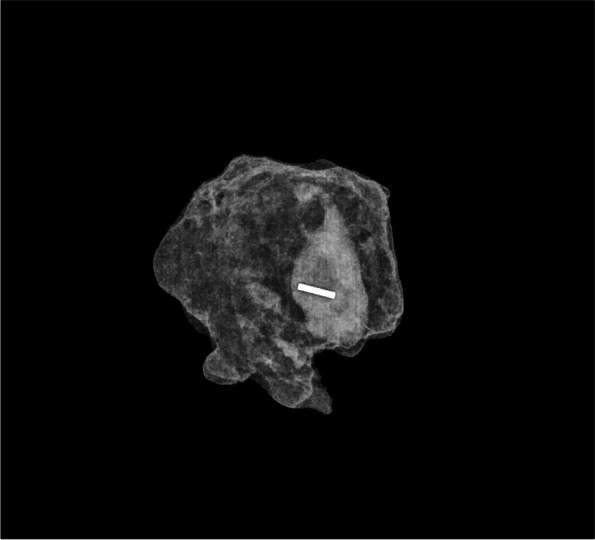
Specimen X-ray of the magnetic seed introduced in the lymph node (The Institute for the Care of Mother and Child)

The magnetic seed is using a magnetic susceptibility for its localisation without any radiation restrictions. The main disadvantages are the need for frequent probe Sentimag recalibration due to the interference with paramagnetic instruments and the limitation of the marker implantation up to 30 mm by a manufacturer [2]. Gabrielová et al. published an in vitro analysis of various localisation markers with magnetic seed reliably detected only up to 2 cm compared to iodine seed with a signal up to 6 cm [14]. This information could be crucial for the detection of deeper lesions. Our results confirmed that magnetic seed could be detected up to 2.5 cm without complications during detection. Deeper lesions could be also detected by probe palpation by pushing the probe on the tissue, thus lowering the probe to marker distance [2]. Lymph node localisation in breast cancer patients by a magnetic seed is quite a new approach, so only a few studies have been published. Martínez et al. (MAGNET study) recruited 81 patients for pathological lymph node marking before NAC by a magnetic seed with an identification rate during the surgery of 100% proving its reliability [4]. Our experiences confirmed the results of the above-mentioned study, magnetic seed was a reliable marker for long-term lymph node marking in our cohort (Fig. 2).

**Fig. 3:**
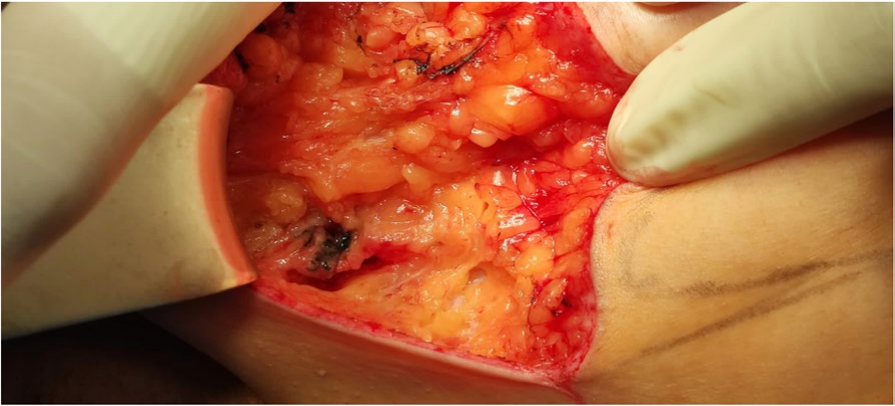
Incision in the axilla with visualised targeted lymph node by a carbon suspension (Silesian Hospital in Opava)

**Fig. 4:**
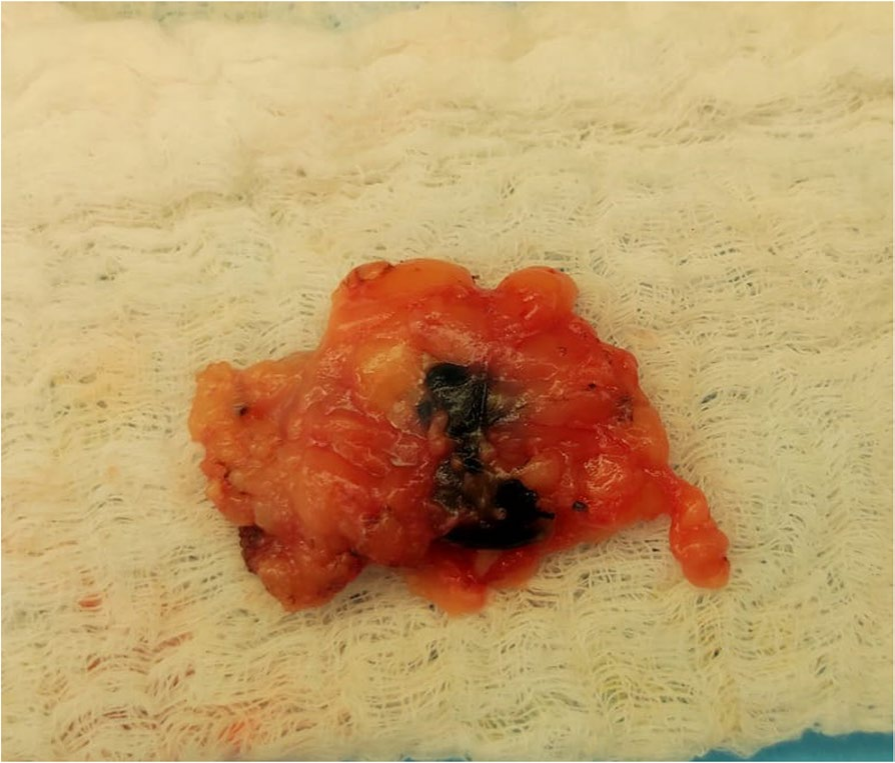
Excised carbon suspension targeted lymph node (Silesian Hospital in Opava)

**Fig. 5:**
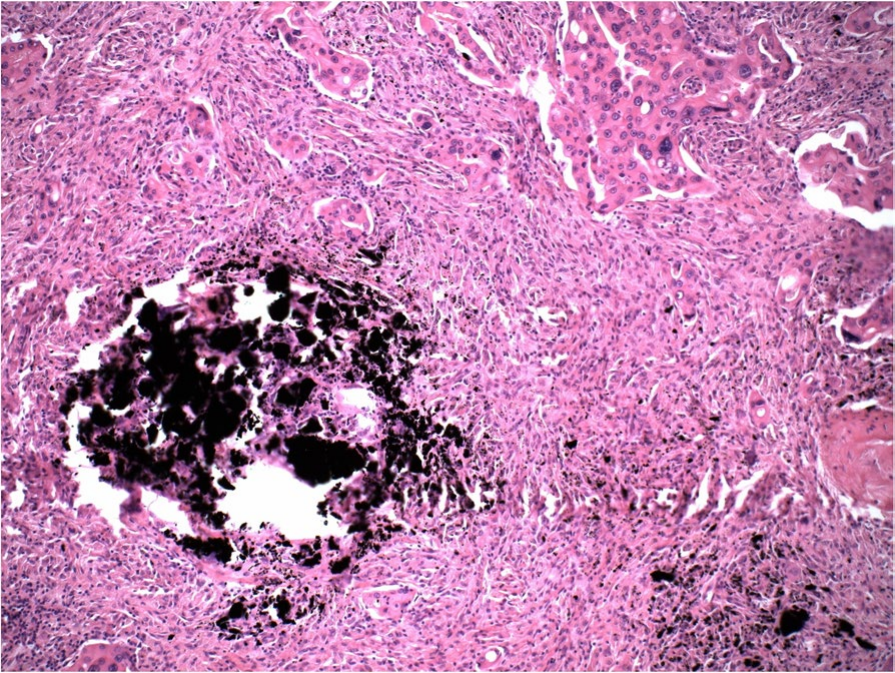
Histopathological examination of carbon suspension targeted lymph node (Department of Pathology, Silesian Hospital in Opava; HE, × 100)

Carbon suspension localisation is the cheapest method of all studied localisation techniques in our cohort, but few authors already proved the reliability and safety of this method for lymph node localisation [6, 15, 16]. In comparison to localisation systems with probes, the carbon suspension needs a larger incision due to only visual navigation, so the surgeon needs to visualize the whole axilla with lymph nodes (Figs. 3 and 4). We suggest, that the radiologist can create a small way by carbon suspension from the targeted lymph node to the skin for more precision localisation and therefore surgeon can operate according to this way with a smaller incision. The carbon suspension creates a black pigment in the lymph node (Fig. 5) examined by a pathologist causing a nonspecific granulomatous reaction, but the quality of histopathological examination is not affected [5, 15]. The carbon suspension disadvantages are mainly non-visibility under an ultrasound, so the radiologist cannot decide, which lymph node is the marked one, and a lower accuracy due to the detection without a probe.

Marker dislodgement is one of the main monitored parameters for marker reliability. Magnetic seed dislodgement was published by a few authors only as a rare case with an incidence of 2.4 or 2.2% [2, 17]. In comparison iodine seed dislodgement is listed in literature also rarely; Barentsz et al. published a review with iodine seed dislodgement between 0 and 0.6% of cases [18]. Clip dislodgement was described by multiple authors [1, 19]. Caudle et al. performed a routine mammographic axillary examination due to the possibility of clip dislodgement and did not reveal any radiologically dislocated clip, but five patients were excluded from the analysis because the clipped lymph node was not identified in the surgical specimen [1]. Carbon suspension as a type of dye seems not to be possible to migrate, but Natsiopoulos et al. published a study with the possibility of carbon migration from one lymph node to another in 45.3% of patients [7]. Any of the lymph nodes with carbon migration were not identified as targeted nodes during surgery, but histopathological examination revealed a small black pigment [7]. This finding could be informative for pathologists because the carbon-marked lymph node could be identified visually only and not during a histopathological examination.

Apart from the three markers studied in our trial, there are also other markers for pathological lymph node localisation. Baker et al. published a prospective pilot study with a SCOUT® radar localisation system and concluded, that the system is feasible for TAD [20]. Gallagher et al. published a prospective study of 101 patients with a radar localisation for TAD and concluded that the method has high accuracy and feasibility when the marker is placed before NAC [21]. The drawback of this method is the interference with electrocautery with the possibility to damage a reflector causing peroperative detection failure [11]. Another method is the Hologic LOCalizer tags using a radiofrequency-based localisation. Lowes et al. published an analysis with 150 patients and 177 tags introduced mainly in breast tumours, but also with 6 cases of axillary lymph node localisation [22]. Another two authors published studies of TAD with radiofrequency tags proving its feasibility, but both studies were only with a few cases, so a larger prospective trial is needed [23, 24]. The main drawback of SCOUT and LOCalizer is the size of the marker (10 and 12 mm) in comparison to iodine or magnetic seed (4.5 and 5 mm), therefore we suppose, that localising smaller lymph nodes could be challenging. These two localisation methods are not available in most hospitals in the Czech Republic; therefore, we could not use them for the IMTAD study.

Another possible localisation method is the clip implantation before the NAC with wire-guided localisation before the surgery. Hartmann et al. published a prospective single-center feasibility trial with discouraging results—the clipped node identification rate was only 70.8% (17/24 cases) and in 6 patients (6/30 cases) the procedure was not finished due to non-visible clip or problems with wire-guided implantation [25]. On the other hand, Gurleyik et al. proved the feasibility and accuracy of this method by analyzing 64 patients and achieving an identification rate of 98.4% using clip localisation followed by wire-guided localisation [26]. Despite this, there are still disadvantages related to wire-guided localisation such as patient discomfort, wire dislocation or transection, and more difficult time management before the surgery. Therefore, we find non-wire localisation methods more useful.

The incidence of postoperative complication in patients with TAD was 7.8% with mostly seroma and a haematoma in the wound without a statistically significant difference between localisation markers. The incidence of seroma is generally high in breast cancer patients occurring between 2.5 and 90% according to various authors [27, 28]. One case of lymphoedema after TAD occurred in our cohort with an incidence of 0.9%. Lee et al. published a retrospective study comparing TAD and ALND with lymphoedema incidence of 8.5% and 19.3%, respectively [29]. The difference was statistically significant; therefore, we theorize that future studies will confirm lower lymphoedema incidence in TAD.

The limitation of the study is the number of patients in the subgroup with carbon suspension and magnetic seed and differences between subcohorts characterictics. Given these differences, the results should be reproduced with limitations. Further prospective trials comparing more localisation markers for TAD would be beneficial.

## Conclusion

The IMTAD study indicated, that three studied localisation markers (iodine seed, magnetic seed and carbon suspension) are statistically comparable in terms of complications during marker implantation, marker dislodgement and complications during marker detection. However, it is important to note that the results may have limitations due to variations between subcohorts. Iodine seed, magnetic seed and carbon suspension seem to be reliable, accurate and safe markers for pathological lymph node localisation in breast cancer patients with targeted axillary dissection after NAC. The study confirmed, that the FNR of the targeted lymph node was lower than the FNR of the sentinel lymph node proving that the targeted lymph node is a better marker for axillary lymph node status after NAC.

## Data Availability

All data generated or analysed during this study are included in this article. Further enquiries can be directed to the corresponding author.
